# The Sanctuary Within: Development of CD20+ CNS Lymphoma Despite Peripheral B‐Cell Depletion by Rituximab in a Multiple Sclerosis Patient

**DOI:** 10.1155/crnm/1186165

**Published:** 2026-05-26

**Authors:** Mohammad Hossein Harirchian, Seyed Ehsan Mohammadianinejad, Asghar Bayati, Vahid Soleimani, Zahra Sarvestani, Maryam Kaeedi, Sanaz Heydari Havadaragh

**Affiliations:** ^1^ Iranian Center of Neurological Research, Neuroscience Institute, Tehran University of Medical Sciences, Tehran, Iran, tums.ac.ir; ^2^ Department of Neurology, School of Medicine, Shahrekord University of Medical Sciences, Shahrekord, Iran, skums.ac.ir; ^3^ Department of Pathology, Faculty of Medicine, Tehran University of Medical Sciences, Tehran, Iran, tums.ac.ir

**Keywords:** comorbid conditions, lymphoma, multiple sclerosis

## Abstract

**Introduction:**

Patients with multiple sclerosis (MS) are not immune to developing comorbid conditions, including malignant disorders. A new neurologic worsening in a known MS patient, despite being on immunosuppressive or even antineoplastic therapy, could be a malignant condition, other than MS relapse.

**Case Presentation:**

We report a middle‐aged woman with a known history of MS and under treatment with rituximab from a few years ago, who subsequently developed clinical and imaging features that were not typical for MS relapse, which was pathologically confirmed to be a diffuse large B‐cell lymphoma (DLBCL).

**Conclusion:**

It is important to consider the possibility of superimposed comorbid disorders, especially malignant conditions, in the setting of immunosuppressive therapy in MS patients. Considering red flags is an important task before the diagnosis of MS relapse as an explanation for new neurologic worsening.

## 1. Introduction

Multiple sclerosis (MS) is driven by compartmentalized intrathecal inflammation that is limited within CNS. This process is independent from systemic inflammation, which explains why peripherally administered therapies such as rituximab often fail to suppress it [1, 2]. This inflammation correlates with MS severity [1] and contributes to CNS tissue destruction [3], underscoring its central pathogenic role in disease progression.

We report the case of a patient with a known diagnosis of MS who developed new neurological deterioration, where atypical clinical and imaging features ultimately revealed diffuse large B‐cell lymphoma (DLBCL). We highlight the clinical and imaging red flags that can call for a diagnosis other than MS.

## 2. Case Report

**FIGURE 1:**
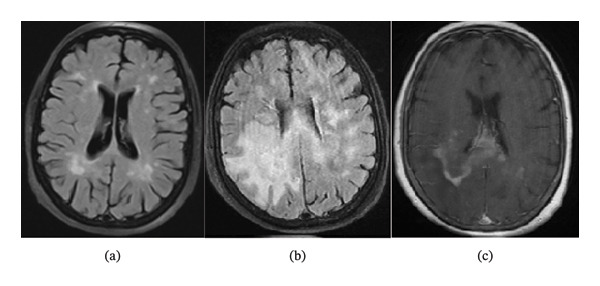
(a) (FLAIR): The patient’s previous typical MS lesions (2017); (b) (FLAIR) and (c) (T1 with contrast): Atypical lesions in recent brain MRI; diffuse large B‐cell lymphoma was confirmed.

The patient is a 58‐year‐old Iranian woman, known case of MS for the last 13 years, who initially presented in 2010 with right optic neuritis and typical demyelinating lesions in the brain and cervical MRI (Figure 1). There was also a family history of MS in her sister. The patient was first treated with Interferon Beta‐1a for 10 years, but subsequently, based on clinical and imaging evidence of disease activity, her treatment was escalated to rituximab in 2019.

She was referred and admitted due to the subacute progressive headache, nausea, and vomiting, followed by imbalance, cognitive problems, and drowsiness that started and progressed about 1 month ago. The patient was not febrile during this period. Upon admission, her initial physical examination revealed impaired attention and disorientation, bilateral papilledema, asymmetric quadriparesis (more prominent on the left side), spasticity, and hyperreflexia with upward plantar reflexes on both sides. She was unable to walk.

**FIGURE 2:**
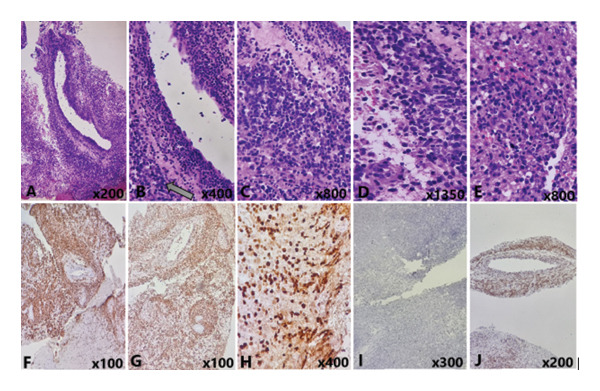
Pathology and IHC assessment. (A) Perivascular infiltration of large atypical lymphocytes. (B) High magnification of large atypical lymphocytes around CNS vessels (arrow). Prominent endothelial cells are seen in the vessel wall. (C) Diffuse infiltration of large lymphocytes in brain tissue. (D) In the left, scattered RBCs, and in the right, neoplastic large lymphocytes, some with vesicular nuclei and one or more nucleoli. (E) Infiltration of large lymphocytes in brain tissue and few interspersed RBCs. (F) IHC staining for CD20 shows diffuse aggregation of neoplastic lymphocytes around vessels and in brain tissue. (G) LCA staining in the IHC study of neoplastic lymphocytes. (H) Ki67 staining in neoplastic lymphocytes. The right side of the image revealed a crushing artifact of lymphocyte nuclei (IHC study with high magnification × 400). (I) BCL6 immunostaining in the IHC study shows negative lymphocytes (nongerminal center type). (J) BCL2 IHC staining in perivascular lymphocytes.

The progressive encephalopathy, worsening headache and vomiting, and evidence of increased intracranial pressure were all strong red flags for the diagnosis of MS relapse. The patient was taking anti‐CD20 as a high‐efficacy MS treatment for more than 3 years, so the probability of superimposed infection or neoplastic process was first considered. Brain MRI (Figure 1) showed extensive confluent abnormal white matter signal intensity in both the centrum semiovale crossing the splenium of the corpus callosum with an extension to the midbrain and the right side of the pons (more dominant on the right side). Mild degrees of mass effect and midline shift were evident, and patchy enhancement and diffusion restriction were seen in parts of the lesions (Figure 2). The MRI features were also red flags for MS relapse and strongly suggested a superimposed neoplastic and less likely an infectious pathology.

Cerebrospinal fluid (CSF) analysis showed high protein (120 mg/dL) and high LDH levels (155 units/L) with a normal glucose level (83 mg/dL). Infectious assessments in CSF, including Gram and acid‐fast staining and PCR of tuberculosis (TB) and viruses, were all reported negative. CSF cytology demonstrated a few atypical lymphoid cells.

Because of the progressive altered consciousness in the initial days of admission and evidence of intracranial hypertension, a 5‐day treatment with intravenous (IV) pulse methylprednisolone was started, which resulted in partial initial improvement.

Regarding the atypical clinical and imaging findings and progressive course of the disease, a diagnostic biopsy of the brain was planned for her. Pathological and immunohistochemistry (IHC) assessments confirmed a high‐grade B‐cell lymphoproliferative disorder consistent with DLBCL (Figure 2). The CD20 marker was reported positive in the majority of mature lymphocytes, and BCL2 was detected in 40% of lymphocytes. The ki67 index showed a proliferation capacity of about 50%–60% of tumor cells in hot spot areas. Based on the Hans algorithm and negative BCL6, the diagnosis of nongerminal center diffuse large B‐cell (non‐GCB) lymphoma was confirmed. The chemotherapy protocol was initiated with a high dose of rituximab and MTR regimen (IV high‐dose methotrexate, oral temozolomide, and IV rituximab). Unfortunately, she did not respond significantly to the treatment and was planned for radiotherapy, which was not possible as she developed septicemia, myocardial infarction, and lower gastrointestinal bleeding. Unfortunately, she passed away 6 months after her first symptoms due to multiorgan failure.

## 3. Discussion

MS is a chronic immune‐mediated disease that requires chronic and often life‐long treatment. It is not rare for MS patients to develop associated immune‐mediated, infectious, or even neoplastic infiltrative disorders [4]. Several studies suggest that individuals with MS may have an altered cancer risk compared to the general population. Grytten et al. suggest a higher likelihood of cancer, particularly hematological cancers [5], while others, such as Etemadifar et al., report lower overall cancer rates except for lymphomas, nervous system malignancies, and breast cancer [6].

Among these neoplastic disorders, primary central nervous system lymphoma (PCNSL) is a rare but aggressive malignancy, representing approximately 2%‐3% of all brain tumors and less than 1% of all non‐Hodgkin lymphomas [7]. Although rare, isolated cases of PCNSL have been documented in MS patients, often during immunotherapy (Table 1) [4, 8–15]. Such cases suggest diagnostic challenges and the potential for disease overlap or transformation.

**TABLE 1:** Reported cases of PCNSL in patients with MS undergoing immunosuppressive/immunomodulatory therapy.

Authors	Age	Sex	Duration of MS (months)	Drugs before lymphoma	CT regimen	Outcome
Lyons et al. [4]	53	Male	About 36	MP, IFN‐β1a, GA	MTX	Died (1 m) after PCNSL diagnosis
Kitazaki et al. [8]	56	Male	4	MP, DMF, IVIG	RTX, MTX, Ara‐C	Improved; discharged with caregiver
Yang et al. [9]	33	Male	36	MP, IFN‐β1a, AZA, MMF	N/S	Died (2 m) due to sepsis
Chiang et al. [10]	61	Female	About 370	MP, IFN‐β1a, IFN‐β1b	MATRIX protocol	Near‐complete resolution on MRI after the 4th cycle of CT
Schweikert et al. [11]	40	Male	About 36	MP, IFN‐β1a, AZA, NTZ	MTX‐based regimen	Favorable 2 y response; no MRI flare
Burgetova et al. [12]	27	Male	About 60	MP	N/S	N/S
Phan‐Ba et al. [15]	40	Male	240	MP, NTZ	N/S	N/S
Matzke et al. [13]	40	female	43	MP, AZA, IFN‐β1a, NTZ	MTX, Ara‐C	Died (3 m) after PCNSL relapse
Na et al. [14]	28	male	48	MITX, NTZ	N/S	Died
Current study	58	female	About 156	IFN‐β1a, RTX	MTR regimen	Died (6 m) after symptom initiation

*Note:* We summarized demographic details, MS duration, treatments received prior to lymphoma diagnosis, chemotherapy regimens, and patient outcomes. MP = methylprednisolone; IFN‐β1a = Interferon Beta‐1a; IFN‐β1b = Interferon Beta‐1b; DMF = dimethyl fumarate; IVIG = intravenous immunoglobulin; AZA = azathioprine; MMF = mycophenolate mofetil; NTZ = natalizumab; RTX = rituximab; MTX = methotrexate; Ara‐C = cytarabine; TTP = thiotepa; MITX = mitoxantrone; MATRIX protocol = RTX + MTX + Ara‐C + TTP; MTR = MTX + TMZ (temozolomide) + RTX; CT = chemotherapy.

Abbreviations: GA = glatiramer acetate; N/S = not specified.

In this context, we present a case of a patient diagnosed with relapsing‐remitting multiple sclerosis (RRMS) receiving rituximab, who under follow‐up, exhibited significant clinical deterioration and atypical MRI features for an MS attack leading to the diagnosis of non‐GCB. To our knowledge, this represents the first reported case of DLBCL developing in an MS patient during long‐term rituximab therapy.

Rituximab, a monoclonal anti‐CD20 antibody, is a potent disease‐modifying treatment (DMT) widely used off‐label for MS. Interestingly, it is also considered as one of the first‐line chemotherapeutic drugs for systemic DLBCL patients or R‐CHOP (rituximab, cyclophosphamide, doxorubicin, vincristine, and prednisone) and a part of MTR regimen (intravenous high dose of methotrexate, oral temozolomide, and intravenous rituximab) in CNS DLBCL (as used in our patient too) [16]. However, because of the large molecular size, it is a matter of debate whether it passes the BBB and has favorable effects on PCNSL treatment [17].

Additionally, given the immunomodulatory effects [18, 19], investigations took place regarding the potential long‐term risk of malignancies in MS patients undergoing rituximab therapy. A nationwide research in Sweden found no increased risk of cancer in the patients treated with rituximab compared to those receiving other DMTs and general population [20]. Similarly, a recent meta‐analysis reported an “ignorable” pooled prevalence (1 in 100,000) of cancer in MS patients receiving rituximab therapy [21]. Also, other reports concluded that any observed associations were more likely incidental rather than causal [22]. Collectively, these reports support our patient’s exceptional presentation.

Moreover, the potential mechanisms by which immunosuppressive/immunomodulatory therapies (such as rituximab) may contribute to cancer incidence in MS patients are complex. While these medications are effective at controlling disease activity, they may interfere with immune surveillance (the natural ability to detect and remove abnormal or precancerous cells) [23]. In our patient, rituximab’s limited BBB penetration [24] may permit CNS B‐cell clones to persist unchecked, effectively creating a “sanctuary site,” while peripheral B‐cells remain depleted. Also, CD20+ B‐cells may have migrated into the CNS during rituximab therapy while evading peripheral depletion, though we note that CD19+/CD20+ cell counts were not monitored during the last hospitalization to confirm the degree of B‐cell depletion. However CD19+/CD20+ blood cell counts were routinely monitored via flow cytometry in outpatient follow‐ups during patient’s rituximab therapy to guide rituximab administration (maintained below 1%) [25]. Long‐term immunosuppression may also disrupt immune homeostasis, leading to chronic inflammation, impaired apoptosis, or errors in DNA repair, all of which may increase the risk of genetic instability [26].

## 4. Conclusion

MS is a chronic inflammatory and degenerative immune‐mediated disease. Patients receiving potent immunosuppressive therapies may be at increased risk of superimposed infectious or neoplastic comorbidities over time. Despite the observations in this study, there is currently no conclusive evidence establishing a direct association between MS, rituximab, and the development of PCNSL.

**Nomenclature**
MS: Multiple sclerosis
CNS: Central nervous system
MRI: Magnetic resonance imaging
CSF: Cerebrospinal fluid
PCR: Polymerase chain reaction
TB: Tuberculosis
IV: Intravenous
IHC: Immunohistochemistry
DLBCL: Diffuse large B‐cell lymphoma
LDH: Lactate dehydrogenase
non‐GCB: Nongerminal center B‐cell
DMT: Disease‐modifying treatment
RRMS: Relapsing‐remitting multiple sclerosis
PCNSL: Primary CNS lymphoma

## Author Contributions

Asghar Bayati was involved in visiting and diagnosing the patient and also in writing the first draft of the manuscript. Vahid Soleimani reviewed the biopsy samples of the patient and the pathologic confirmation of the diagnosis. Zahra Sarvestani, Maryam Kaeedi, and Sanaz Heydari Havadaragh were involved in writing the manuscript and its conception. Mohammad Hossein Harirchian and Seyed Ehsan Mohammadianinejad revised the manuscript. Finally, Sanaz Heydari Havadaragh submitted the manuscript.

## Funding

The authors received no financial support for the search, authorship, or publication of this manuscript.

## Disclosure

All authors reviewed and edited the manuscript and approved the final version.

## Ethics Statement

This study was conducted under the Declaration of the Helsinki protocol. The patient’s legal guardian has given written informed consent to contribute to this study.

## Consent

Written informed consent was obtained from the patient for publication of this case report and any accompanying images. A copy of the written consent is available for review by the Editor‐in‐Chief of this journal.

## Conflicts of Interest

The authors declare no conflicts of interest.

## Data Availability

The data that support the findings of this study are available from the corresponding author upon reasonable request.
